# Rhupus Syndrome: A Diagnostic Dilemma

**DOI:** 10.7759/cureus.29018

**Published:** 2022-09-11

**Authors:** Susmita Upadhyaya, Mayank Agarwal, Ashutosh Upadhyaya, Monika Pathania, Minakshi Dhar

**Affiliations:** 1 Internal Medicine, All India Institute of Medical Sciences, Rishikesh, Rishikesh, IND; 2 General Physician, Evergreen Hospital Pvt. Ltd, Parasi, NPL

**Keywords:** rheumatoid arthritis, erosive polyarthritis, autoimmune, rheumatic, serositis, renal involvement, hematological, sle, rhupus

## Abstract

Rhupus syndrome, a rare coexistence of systemic lupus erythematosus (SLE) and rheumatoid arthritis, is characterized by symmetrical erosive polyarthritis and permanent deformities in addition to the clinical and serological characteristics of SLE. Its prognosis is further complicated by neurological and hematological involvement, which dramatically lowers patients' perceptions of their quality of life in terms of their health. Rhupus individuals have significantly less kidney involvement than SLE patients do. We present a case of a young female who had symmetric, bilateral, erosive polyarthritis for one and a half years preceding the signs and symptoms of SLE, which occurred about six months later.

## Introduction

There are more than 200 ailments classified as rheumatic diseases, multisystem inflammatory disorders defined by immunological dysregulation that typically impact the bones, muscles, and joints. Clinically, they can be identified by pain, inflammation, stiffness, deformity, and various levels of disability [1].

Although systemic autoimmune disorders have defined diagnostic criteria and guidelines, up to 25% of patients with these symptoms go misdiagnosed and develop full-blown disease, with flares and complications brought on by incomplete or delayed treatment. Having multiple rheumatic diseases in one person makes the diagnosis more challenging. Several types of overlap syndrome are documented in the literature. While two or more conditions may coexist, one disease often has the clinical upper hand. Rhupus syndrome, which combines the clinical and laboratory aspects of rheumatic arthritis (RA) and systemic lupus erythematosus (SLE), is one of the uncommon and sporadically documented overlaps. SLE and RA are systemic autoimmune rheumatic illnesses that affect multiple organs and systems and have unique clinical and serological traits [2].

The term Rhupus was used for the first time in the year 1971 to refer to this condition. The prevalence of Rhupus syndrome has been estimated to be around 0.01% to 2% of patients with rheumatic diseases [1,2]. It has been defined as a deforming and erosive symmetric polyarthritis accompanied by symptoms of SLE and the presence of antibodies such as anti-double stranded DNA (anti-dsDNA), anti-Smith, and rheumatoid factor (RF) with or without anti-cyclic citrullinated peptides (anti-CCP) antibody [2,3]. Typical arthritis associated with SLE is rarely erosive and differs from RA [3,4]. Unlike SLE patients, Rhupus patients have significantly less kidney involvement, while no differences have been observed between neuropsychiatric, cutaneous, and hematological involvement or serositis [4].

Given the rarity of occurrence and dearth of publications on this illness, particularly in India, we provide a case of a young female who presented with a fever of unknown origin and polyarthralgia and was eventually diagnosed with Rhupus syndrome.

## Case presentation

A young unmarried female with no prior comorbidities presented with a six-month history of intermittent low-grade fever with episodes of high-grade fever spikes, documented up to 102°F. She also had complaints of dry cough and shortness of breath that gradually progressed from modified Medical Research Council (MMRC) Grade I to MMRC Grade II associated with bilateral pleuritic chest pain. There was no history of orthopnea, syncope, hemoptysis, yellowish discoloration of eyes, abdominal pain, decreased urine output, and burning micturition. Evaluation for tropical infections and pulmonary tuberculosis had been negative. However, she had received multiple courses of empirical antibiotics with no improvement. She presented to our hospital with her symptoms, persistent and undiagnosed.

**Figure 1:**
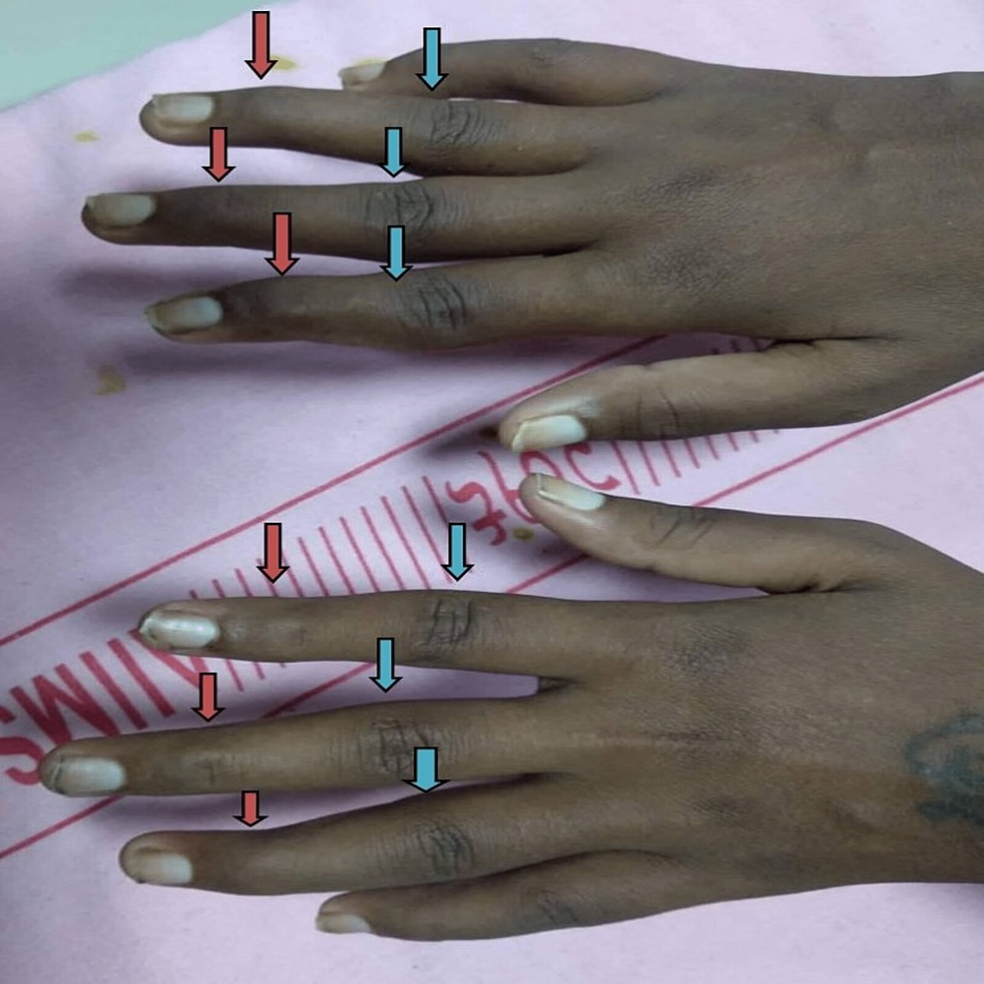
Hyperextension deformity in the proximal interphalangeal joints (blue arrow), flexion deformity in the distal interphalangeal joints (red arrow) in both hands.

**Figure 2:**
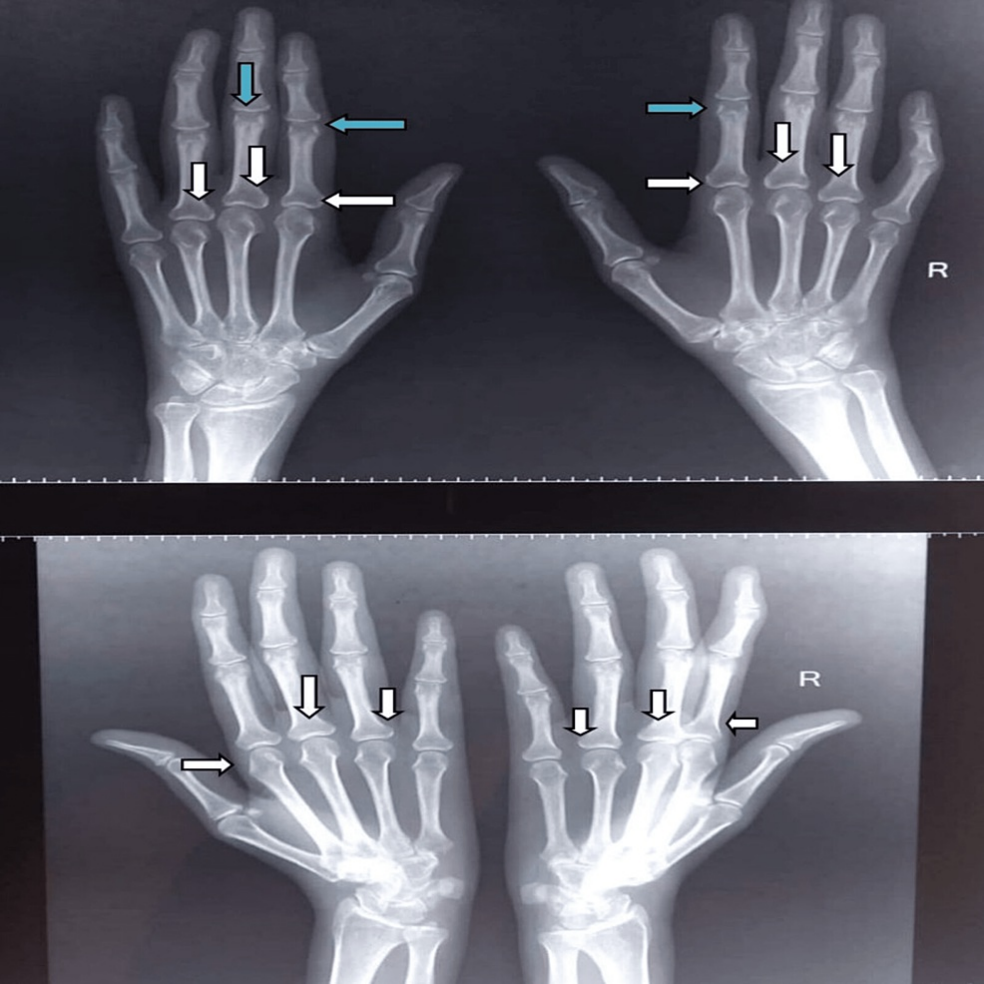
X-ray showing juxta-articular osteopenia and periarticular erosions in proximal interphalangeal joints (blue arrow) and metacarpophalangeal joints (white arrow).

**Figure 3:**
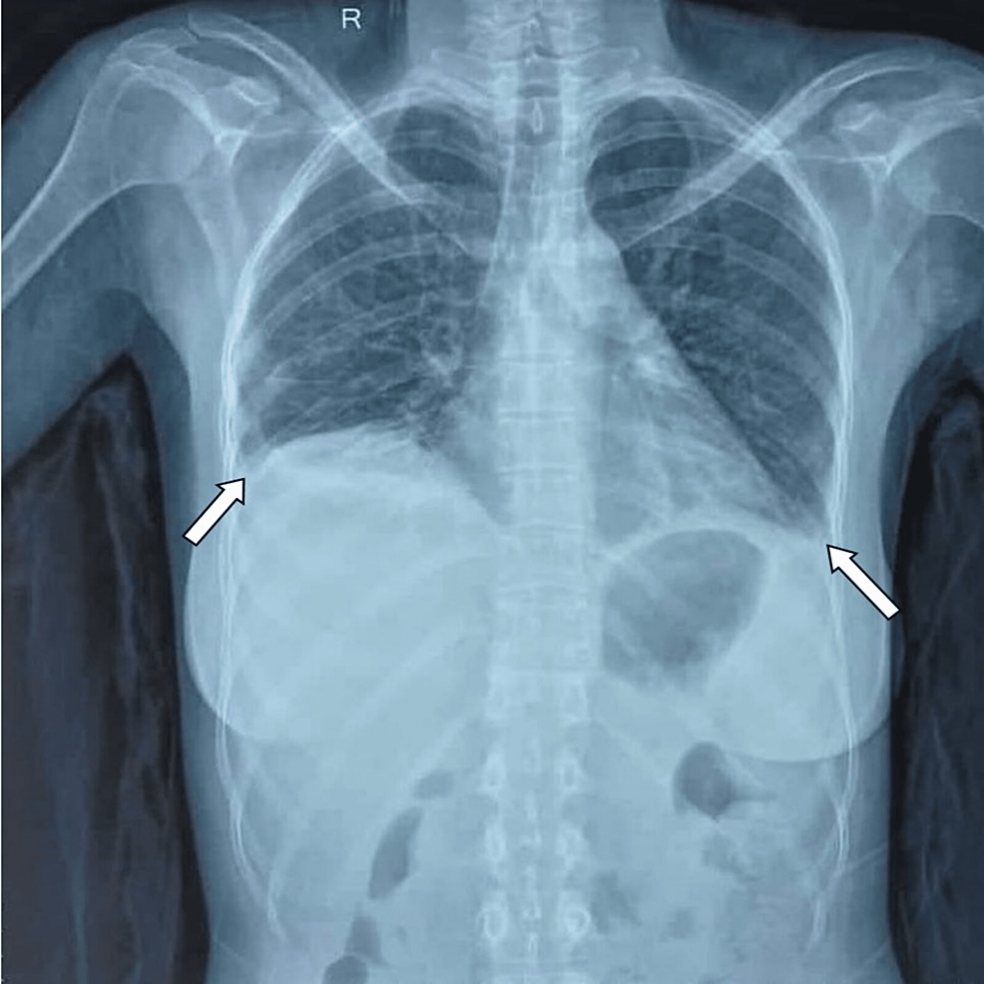
Chest x-ray suggestive of bilateral pleural effusion (white arrow).

On examination, she was tachypneic, pale, and had non-scarring alopecia. Upon auscultation, there was decreased air entry in bilateral infra-scapular and infra-axillary regions. On musculoskeletal examination, the patient had hyperextension deformity in the proximal interphalangeal (PIP) joints, hyperflexion deformity in the distal interphalangeal (DIP) joints on bilateral hands along with ulnar deviation of the bilateral fingers (Figure 1). There was a limitation in the flexion/extension of both wrists (30°). X-rays showed bilateral juxta-articular osteopenia and per-articular erosions of PIP and DIP joints (Figure 2). Chest-ray showed bilateral pleural effusion (Figure 3). Her lab parameters showed anemia, neutropenia, thrombocytopenia, and raised inflammatory markers. Cultures were sterile. Serum procalcitonin was within normal limits. A contrast-enhanced computed tomography (CECT) of the thorax and abdomen was obtained to evaluate fever of unknown origin that revealed bilateral pleural effusion, ascites, and multiple enlarged retroperitoneal and mesenteric lymph nodes. Upon further evaluation, the Anti-nuclear antibody (ANA) test via indirect immunofluorescence was positive for 1:640 titers. Her anti-dsDNA antibody was positive along with a low complement level (C3, C4), suggesting SLE as per 2019 European League Against Rheumatism/American College of Rheumatology criteria (Table 1).

RF and anti-CCP antibodies were high-positive. On reviewing the history, the patient recalled having migratory joint pain involving both small and large bilateral upper limb and lower limb joints for one and half years. Taking into account the clinical and immunological profile of the patient, she was diagnosed as a case of SLE with a rheumatoid overlap - Rhupus syndrome. The patient was prescribed corticosteroids, hydroxychloroquine, and methotrexate to manage the disease and prevent its progression at the time of discharge and was advised for regular follow-up.

**Table 1:** Serology RF: Rheumatoid factor; anti-CCP antibody: anti-cyclic citrullinated peptide antibody; ANA: Anti-nuclear antibody; IFA: Immunofluorescence assay; anti-dsDNA antibody: anti-double-stranded DNA antibody; anti-SSA antibody: anti–Sjögren's syndrome-related antigen A antibody; anti-SSB antibody: anti–Sjögren's syndrome-related antigen B antibody.

Serological tests	Values	Reference values
RF, quantitative	27.9 IU/mL	<15 IU/mL
Anti-CCP antibody	>195 EU/mL	<20 EU/mL
ANA/IFA	Positive	
ANA primary dilution	1:80	
ANA primary intensity	4+	
ANA pattern	homogenous	
ANA endpoint titer	1:640	
Anti-ds DNA antibody	Positive	
Anti-SSA antibody	Positive	
Anti-SSB antibody	Positive	
Anti-nucleosome antibody	Positive	
Anti-histone antibody	Positive	

## Discussion

Rarely, RA and SLE coexist to cause Rhupus syndrome. Given the absence of specific criteria that describe this entity, it is challenging to identify such patients [2]. The syndrome is mostly reported as being more common in women and, in most cases, beginning with RA-like symptoms before progressing to SLE. Conversely, reports of concurrent symptoms and vice versa are uncommon. According to reports, the clinical signs of SLE often begin between 4 and 7 years after the patient develops RA [1,5]. For one and a half years, our patient had symmetric, bilateral, erosive polyarthritis that went undetected and untreated. After about six months, SLE symptoms started to appear.

The most reported clinical features of Rhupus Syndrome are erosive polyarthritis, rheumatoid nodules, malar rash, photosensitivity, alopecia, and the presence of constitutional symptoms. Renal and neurological involvement is rarely reported [2,4]. In our patient, erosive polyarthritis appeared as the initial symptom of RA. In contrast, the manifestations of SLE, chronic fever, pleuritic chest pain, cytopenia, polyserositis, and non-scarring alopecia were those of greater clinical significance. There were no other complications related to the underlying diseases, such as the presence of neurological or kidney involvement or rheumatoid nodules. It is described that different types of joint manifestations can occur in the clinical course of SLE, including the more frequent appearance of arthralgias and non-erosive polyarthritis with reversible deformities [3,4]. As a result of lax joint capsules, tendons, and ligaments, metacarpophalangeal (MCP) subluxation, ulnar deviation, and swan neck deformities are typical SLE findings [3]. Swan neck abnormalities and ulnar deviation were observed in our patient.

The outcomes of the diagnostic procedures are significant in making the final diagnosis of the illness. It has been discovered that the genes programmed cell death 1 (PDCD1), signal transducers and activators of transcription 4 (STAT4), Fc-receptor-like 3 gene (FCRL3), and protein tyrosine phosphatase nonreceptor 22 (PTPN22) are related to RA and SLE. Some studies have shown that Rhupus patients have significantly higher levels of the human leukocyte antigen (HLA)-DR1 and HLA-DR2 alleles [6-8]. Financial limitations prevented our patient from having the test done.

Anti-CCP antibody has been utilized to distinguish RA and Rhupus from SLE [1,2]. In our case, the presence of anti-dsDNA and anti-CCP antibodies confirms that Rhupus syndrome is an overlap disease rather than a subtype of SLE. Symmetric and bilateral erosive joint patterns were seen in imaging studies of our patient. The symmetric bilateral erosive polyarthritis is part of the 2020 EULAR/ ACR diagnostic criteria for RA. Anti-CCP antibodies may have a pathogenic role in the emergence of substantial erosions, according to Chan and colleagues. They suggested that anti-CCP antibody-positive SLE patients are more likely to have erosive arthritis [6,9]. The polyarticular, erosive, and seropositive inflammatory picture justifies the application of hydroxychloroquine, corticosteroids, and methotrexate to control the articular inflammatory process and alleviate constitutional symptoms. Other medications that can be helpful, especially if there is evidence of renal involvement, are mycophenolate mofetil, and biologic therapy. All tumor necrosis factor-alpha (TNF-alpha) inhibitors have been linked to the onset of ANA, anti-dsDNA, and drug-induced lupus, including confirmed SLE renal disease. Thus, when methotrexate fails to control erosive RA adequately, introducing biologic therapy requires monitoring for aggravation and new onset symptoms of SLE [2,3]. The clinical signs of this overlap disease should be continuously evaluated to help avoid consequences.

## Conclusions

Rhupus syndrome, an overlap of RA and SLE, has variable clinical and serological characteristics. As the erosive joint injury progresses, more articular tissue is destroyed, increasing the degree of impairment. Despite being an uncommon condition, understanding its diverse clinical presentation and diagnostic features is crucial for prompt diagnosis, avoiding delayed treatment, and minimizing sequelae. It is necessary to conduct more research on the renal involvement of this overlap phenomenon. There is a need for definite, unambiguous guidelines for diagnosing Rhupus syndrome.
